# Variability in exercise is linked to improved age-related dysfunctions: A potential role for the constrained-disorder principle-based second-generation artificial intelligence system

**DOI:** 10.21203/rs.3.rs-3671709/v1

**Published:** 2023-12-19

**Authors:** Yaron Ilan

**Keywords:** Aging, disorder, artificial intelligence, variability, defective engineering, complex systems

## Abstract

**Objective:**

Regular physical activity (PA) promotes mental and physical health. Nevertheless, inactivity is a worldwide pandemic, and methods to augment exercise benefits are required. The constrained disorder principle (CDP) characterizes biological systems based on their inherent variability. We aimed to investigate the association between intra-individual variability in PA and disability among non-athlete adults.

**Methods:**

In this retrospective analysis of the longitudinal SHARE survey, we included non-disabled adults aged >50 with at least six visits over 14 years. Self-reported PA frequency was documented bi- to triennially. *Low PA* intensity was defined as vigorous PA frequency less than once a week. *Stable PA* was described as an unchanged PA intensity in all consecutive middle observations. The primary outcome was defined as a physical limitation in everyday activities at the end of the survey. Secondary outcomes were cognitive functions, including short-term memory, long-term memory, and verbal fluency.

**Results:**

The study included 2,049 non-disabled adults with a mean age of 53 and 49.1 % women. In the initially *high PA* intensity group, variability in PA was associated with increased physical disability prevalence (23.3% vs. 33.2%, *stablevs. unstablePA*; P<0.01; adjusted P<0.01). In the initially *low PA* intensity group, variability was associated with a reduced physical disability (45.6% vs. 33.3%, *stablevs. unstable PA;* P=0.02; adjusted P=0.03). There were no statistically significant differences in cognitive parameters between the groups. Among individuals with the same low PA intensity at the beginning and end of follow-up, variability was associated with reduced physical disability (56.9% vs. 36.5%, *stablevs. unstablePA;* P=0.02; adjusted P=0.04) and improved short-term memory (score change: −0.28 vs. +0.29, *stablevs. unstable PA;* P=0.05).

**Conclusion:**

Incorporating variability into PA regimens of inactive adults may enhance their physical and cognitive benefits.

## Introduction

Genetics, the environment, and social factors all contribute to biological aging. A primary objective and global challenge in public health is preserving functional health and quality of life in old age^1^. Sedentary behavior, one of the aging characteristics, causes physiological and structural impairments^2^. The potential role of physical activity in halting aging is a subject of numerous studies. Moderate exercise is suggested to minimize the physiological effects of sedentary lifestyles and increase active life expectancy by preventing chronic diseases and disabling conditions^3, 4^. Physical activity and exercise are believed to be superior to other optimal aging facilitators. However, it is difficult to draw conclusions based on trial results because of inter- and intra-individual differences among aged subjects, heterogeneity of assessment tools, and difficulties implementing trial results in real-world conditions^1^.

Physical activity (PA) plays a pivotal role in maintaining overall health and reducing the economic burden on the healthcare system^5^. Engaging in regular PA reduces the risk of chronic diseases (e.g., hypertension, type-2 diabetes, coronary heart disease, certain types of cancer) and premature mortality^5, 6^. PA is closely linked to mental health, as it can reduce stress, anxiety, and depression and prevent cognitive decline^5, 7^. The World Health Organization (WHO) guidelines define good PA for adults as >150 minutes of moderate-intensity weekly activity^8^. However, everyday social, personal, and medical factors may limit the time, capability, or will to perform PA. According to the 2022 WHO global status report on PA, 27.5% of the world’s adult population (1.4 billion people) do not meet the recommended levels of PA^5^. The prevalence of physical inactivity rises to 36.8% in high-income countries and is more pronounced in women, adolescents, and older adults^5^. Thus, inactivity is widespread, and methods to enhance exercise benefits under these circumstances are required.

The benefits of physical activity for cognitive function in older adults have been proposed. An analysis of all published randomized controlled trials comparing aerobic physical activity programs with any other or no intervention was conducted recently. Participants older than 55 years of age were eligible for inclusion. There was an increase in cardiorespiratory fitness and cognitive capacity in the intervention group in 8 out of 11 studies that involved aerobic exercise interventions. The improvement in cardiorespiratory fitness also correlated with improvements in cognitive capacity. The majority of comparisons, however, did not yield significant results. There is insufficient evidence to show that improvements in cardiovascular fitness are responsible for improvements in cognitive function^9^.

The constrained disorder principle (CDP) characterizes biological systems based on their inherent noise^10^. Biochemical processes, genomes, cellular organelles, and entire organ functions are all characterized by variability^11–23^. As per the CDP aging is associated with changes in systems’ degree of variability and complexity. The aging process can be defined as a reduction in variability or moving beyond the bounds of variability^24^. CDP-based second-generation artificial intelligence (AI) systems are being developed for implementing personalized variability signatures to improve the efficacy of interventions^25–28^. Recently, CDP-based second-generation AI systems were described for improving anti-aging methods. The platform uses constrained noise to enhance system efficiency and slow aging processes^24^.

For professional athletes, intra-individual variability in sports intensity may assist in optimizing performance by targeting different physiological aspects. This variability helps to prevent plateaus and maximizes athletic potential^29–31^. Moreover, constantly high-intensity training may lead to burnout, fatigue, and increased injury susceptibility^32–34^. Therefore, varying intensities in sports training may reduce the risk of overuse injuries and improve long-term performance. Furthermore, incorporating diversity into the training regimen may enhance the psychological aspects of sports performance by maintaining motivation and enjoyment.

Associations between exercise intensity and well-being were demonstrated in non-athletes. Several studies suggested moderate to vigorous training promotes physical health, while light PA may be associated with more significant psychological benefits^35–40^. However, whether or not intra-individual variability in sports habits (i.e., irregular and diverse exercise frequencies, durations, intensities, and types over time) affects health outcomes is not well-studied.

Loss of personal complexity in PA performance is linked to frailty and mortality, and it was previously hypothesized that introducing intra-individual variability into PA programs may improve their anti-aging qualities^24, 41–43^.

The present study aimed to determine the role of variability in physical activity in older subjects to improve functionality by investigating the association between intra-individual variability in PA frequency and disability among non-athlete adults. We have analyzed a large dataset to assess the importance of physical activity variability in mitigating physical and cognitive disability.

## Methods

### Study design, population, and data collection

Data were extracted from the Survey of Health, Aging, and Retirement in Europe (SHARE) - a widely-known, nationally representative longitudinal study of community-dwelling individuals 50 years and older and their spouses in 28 European countries and Israel, initiated in 2004. Data collection was based on computer-assisted interviewing. Additional information about the SHARE study has been published elsewhere^44, 45^. SHARE was approved by the Ethics Committee of the University of Mannheim during the first to fourth waves and by the Ethics Council of the Max Planck Society after that. We used data from the SHARE waves 1 to 8 (2004–2020), excluding the retrospective wave 3 (SHARElife)^46–52^. To allow adequate and representative intra-individual variability with similar functional baseline status in our analysis, we included individuals aged 50–70 who had paid employment at baseline, participated in 6 or 7 prospective waves, and had no disability upon trial enrollment (n = 2281). To maintain balanced sex groups, participants were sampled in a ratio of 1:3 to the “stable” and “unstable” groups (see definitions at *independent variables)*, respectively, across sex strata, resulting in a sample size of 2,049 subjects from 10 countries.

**Figure 1:**
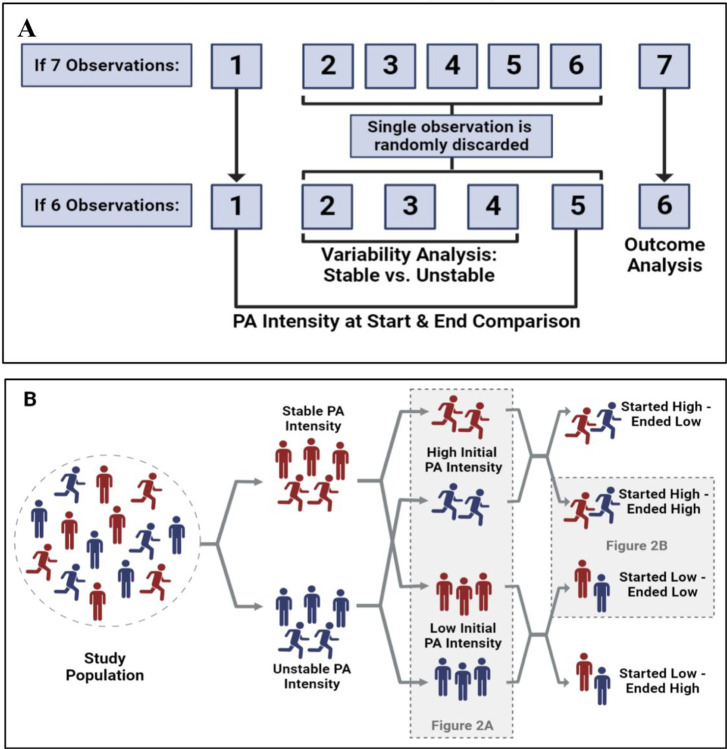
A schematic representation of the study design. (A) The use of the SHARE waves for data analysis. A total of 6 waves for each individual were included. Wave 1 represents the first wave of participation in the cohort (baseline wave). For individuals who participated in all 7 waves, one observation (excluding the first and last ones) was randomly discarded. We used the responses from the first 5 waves included for the intensity analysis of PA (Physical Activity). The final wave was not included in the analysis of the independent variables (PA intensity) but was used for outcomes measurement (disability). (B) **Illustrative flow chart of subgroup allocation**. *Low PA* i ntensity is a vigorous PA frequency of less than once a week. *High PA* intensity is a vigorous PA frequency once a week or more. *Stable PA* intensity is defined as an unchanged PA-reported intensity. *Unstable PA* is defined as a minimum of one change in PA intensity.

Figure 1 outlines the main steps of study design and data analysis. Wave 1 represents the entry wave to the participant cohort (initial). The last wave of each participant - the sixth or seventh - was used to evaluate the outcome. A total of 6 waves for each individual were included in the final analysis. For individuals who participated in all prospective seven waves, one observation (excluding the first and last ones) was randomly omitted for standardization purposes, resulting in 5 input waves (including baseline) and one outcome wave.

#### Variables and Outcomes definitions

##### a. Independent variables:

PA intensity was assessed by presenting a multiple-choice question: “How often do you engage in vigorous PA, such as sports, heavy housework, or a job that involves physical labor?”. There were four optional answers: 1) More than once a week, 2) Once a week, 3) One to three times a month, 4) Hardly ever, or never. *Low PA* intensity was defined as a vigorous PA frequency less than once a week (answers 3 or 4). *High PA* intensity was a vigorous PA frequency once a week or more (answers 1 or 2). We used the responses from the first five waves of each individual for PA intensity analysis. To avoid the effect of transient physical states implicating both physical activity and disability, the final wave of PA intensity assessment of each subject, in which the disability outcomes were measured, was omitted, better reflecting the long-term association between these variables. Intra-individual variability in PA intensity was determined by comparing the sequential personal responses of the three middle-included waves. *Stable PA* intensity was defined as an unchanged PA-reported frequency. *The unstable PA* intensity definition was met if a minimum of one change in PA frequency was reported.

Figure 1B depicts the allocation of the study population into subgroups according to *PA* characteristics along the follow-up.

##### b. Dependent variables/ outcomes

Primary outcome - physical disability: Activities of daily living (ADL) scores related to basic personal care are not expected to identify subtle physical disabilities among adult individuals with working capacities. Therefore, physical disability was evaluated by reported limitations in the functional capacity to perform activities of daily living using a modified ADL score composed of the following ten everyday skills: walking 100 meters, sitting for about two hours, getting up from a chair after sitting for long periods; climbing several flights of stairs without resting; climbing one flight of stairs without resting; stooping, kneeling, or crouching; reaching or extending arms above shoulder level; pulling or pushing large objects like a living room chair; lifting or carrying weights over 10 pounds/5 kilos; and picking up a small coin from a table. A restriction in the performance of any of these activities anticipated to last more than three months by the interviewee was defined as a physical disability.

Secondary outcome - cognitive disability: Three aspects of cognitive function were evaluated: short-term memory, long-term memory, and verbal fluency. For the short-term memory (immediate recall) assessment, the interviewee was asked to repeat a 10-word list immediately. This request was unanticipatedly repeated several minutes later for long-term memory (delayed recall) evaluation. The score in both cases is the sum of recalled words within one minute. Verbal fluency was assessed by requesting the interviewee name as many different animals in one minute. The score is the sum of acceptable animals mentioned.

#### c. Covariates

Age, gender, and diagnosis of chronic diseases at baseline (diabetes mellitus, cerebrovascular accident, ischemic heart disease, and chronic lung disease) were assessed using a self-reported questionnaire.

### Statistical analyses

Continuous variables are presented as means ± standard deviations, and categorical variables are presented as total count and percent of totals. Differences between groups are expressed as absolute values unless specified otherwise.

Comparisons of cognitive scores were done by computing the numerical difference between baseline and end of follow-up for each score. To detect differences between stable and unstable PA intensity groups, t-tests were used for parametric variables, while nonparametric variables were analyzed using the Wilcoxon signed-rank test.

Pearson’s Chi-squared test compared differences in physical disability prevalence. Multivariate analysis and adjustments for possible confounders were made using generalized linear regression models, adjusted for age, gender, and history of diabetes mellitus, cerebrovascular accident, ischemic heart disease, and chronic lung disease. Differences were considered significant for P < 0.05. Values are reported with 95% confidence intervals (CIs) where appropriate, and all P values are 2-sided. Statistical analyses were performed using R software, version 4.2.2.

### Dataset :

*This paper uses data from SHARE Waves 1, 2, 4, 5, 6, 7 and 8 (DOIs*. 10.6103/SHARE.w1.800, 10.6103/SHARE.w2.800, 10.6103/SHARE.w4.800, 10.6103/SHARE.w5.800, 10.6103/SHARE.w6.800, 10.6103/SHARE.w7.800, 10.6103/SHARE.w8.800) *see Börsch-Supan et al. (2013) for methodological details. The European Commission*, *DG RTD has funded the SHARE data collection through FP5 (QLK6-CT-2001–00360), FP6 (SHARE-I3: RII-CT-2006–062193, COMPARE: CIT5-CT-2005–028857,, FP7 (SHARE-PREP: GA N°211909, SHARE-LEAP: GA N°227822, SHARE M4: GA N°261982, DASISH: GA N283646) and Horizon 2020 (SHARE-DEV3: GA N°676536, SHARE-COHESION: GA N°870628, SERISS: GA N°654221, SSHOC: GA N°823782, SHARE-COVID19: GA N°101015924) and by DG Employment, Social Affairs & Inclusion through VS 2015/0195, VS 2016/0135, VS 2018/0285, VS 2019/0332, and VS 2020/0313. Additional funding from the German Ministry of Education and Research, the Max Planck Society for the Advancement of Science, the US National Institute on Aging (U01_AG09740–13S2, P01_AG005842, P01_AG08291, P30_AG12815, R21_AG025169, Y1-AG-4553–01, IAG_BSR06–11, OGH/_04~064, HHSN271201300071C, RAG052527A) and from various national funding sources is gratefully acknowledged (see* www.share-project.org)^53^.

## Results

### a. Sample characteristics:

The sample characteristics stratified by the degree of variability in PA intensity are summarized in Table 1. Of the 2,049 included subjects, 513 (25%) performed stable PA, i.e., the same PA intensity across follow-up, and 1536 (75%; 1:3 ratio) performed *unstable* PA, i.e., varying PA intensity. 1148 (56%) individuals participated in 6 waves, and 901 (44%) participated in 7.

At baseline, the mean age of the *stable PA* group was 53.5 ± 3.8 years and constituted 291 (57%) men, while the mean age of the *unstable PA* group was 54 ± 4.0 years and constituted 870 (57%) men. The prevalence of each chronic disease at baseline was less than 5% within either group, with no statistically significant between-group differences. Cognitive function parameters were also similar between the groups, as detailed. All subjects had no physical disability at baseline. Individuals of the *stable PA* group tended to be more physically active at baseline compared with individuals who exhibited variable PA regimens (85% vs. 67% *high PA* levels, respectively; P< 0.001). They also exhibited the same PA intensity in the first and last (5th) waves (overall stability) more commonly (75% vs. 33%, respectively; P< 0.001).

### b. Physical activity dynamics across PA baseline levels and physical disability and cognitive functions among the entire cohort

We next assessed the impact of PA baseline intensity and its intra-individual variability during follow-up on physical and cognitive disability among the entire study population.

**Figure 2:**
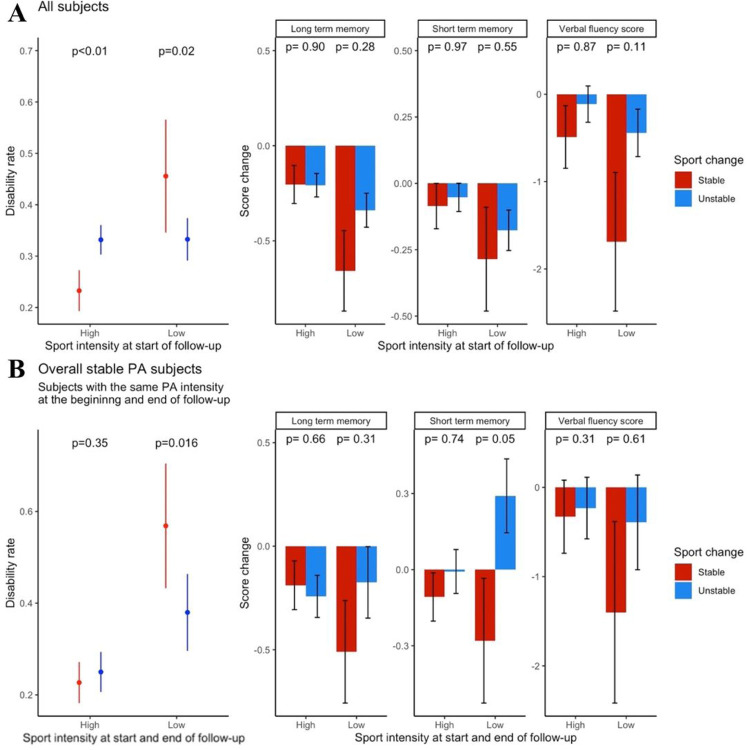
The association between intra-individual variability in PA frequency with physical and cognitive disability (A) Disabilty rates at the end of follow-up (left) and change in cognitive function by the end of follow-up (right) among the entire study cohort across *high* and *low* sport intensity at baseline. (B) Shows a similar analysis as in *A* panel, among the subset of and end of follow-up.

Comparing the physical disability rate in the initially *high PA* intensity group, individuals with *overallstable PA* showed a lower disability rate at the end of follow-up as compared to the *unstable* group (23.3% vs. 33.2% respectively, P< 0.01; OR = 0.6, 95% CI 0.47–0.78). Conversely, in the initially *low PA* intensity group, individuals with *stable PA during follow-up, regardless of the PA status at the end of follow-up*, showed worse disability rates at the end of follow-up (45.6% vs. 33.3%, respectively, P = 0.02, OR = 1.8, 95% CI 1.11 −2.98). Regarding cognitive disability, in both *high* and *low PA* intensity groups, the change in cognitive function parameters was not statistically significant between the *stable* and *unstable PA* subgroups (Fig. 2A(.

### c. Physical activity dynamics on physical disability and cognitive functions among overall stable subjects

We next focused on 891 individuals in our cohort who exhibited identical PA frequencies in the first and last (5th) waves, defined as *overall stable* subjects.

Among them, in the *high PA* intensity group, there was no statistically significant difference between individuals with *stable PA* and *unstable PA* during follow-up regarding physical disability rate (physical disability prevalence was 22.7% vs. 25%, *stablevs. unstable PA* subgroups, respectively, P = 0.35, OR = 0.82, 95% CI 0.58–1.17). However, in the group with *low PA* frequency at baseline and the last follow-up wave, individuals with *stable PA* had a significantly higher physical disability at the end of follow-up (56.9% vs. 36.5%, respectively, P = 0.02, OR = 2.36, 95% CI 1.19–4.73).

Comparing the cognitive functions of individuals with the same PA frequencies in the first last waves - long-term memory and verbal fluency showed no statistically significant differences between individuals with *stable PA* and *unstable PA* in both *high* and *low PA* intensity groups. In the high PA intensity group, short-term memory also showed no statistically significant difference between individuals with stable and unstable PA. Nevertheless, in the *low PA* intensity group, short-term memory improved in the *unstable PA* subgroup compared with the *stable PA* subgroup (−0.28 *stable* group vs. +0.29 *unstable* group, P = 0.05).

## Discussion

The present study’s results support physical activity variability’s importance in improving physical and cognitive outcomes. We used the SHARE dataset to determine the role of variability in physical activity and its association with clinical outcomes. The data shows frequent PA is associated with better physical outcomes, which are enhanced in persistently (non-variable) active individuals. However, among those who performed infrequent PA (i.e., less than once a week), higher intra-individual variability in exercise frequency was associated with less physical disability, even if the last follow-up wave was less intense. This finding implies the importance of introducing variability in the intensity of physical activity among adults with a sedentary lifestyle at an earlier age than 65–70. The end of our follow-up coincides with the retirement age in many SHARE participating countries.

Furthermore, it reinforces the importance of implementing lifestyle intervention programs such as physical activity while the adult remains active in the labor market before retirement age. Workplace intervention programs are beneficial in preserving working capacity, health, and well-being^54^. Further research is needed into increasing PA variability programs targeting older workers over 50 with sedentary lifestyles. Cognitive parameters were generally less affected by variability in PA frequency. Nonetheless, among less active individuals who exhibited the same (low) PA intensity at the start and end of follow-up, higher variability in PA intensity appeared to be related to less short-term memory declines,

Per the CDF, noise is fundamental for the proper function of biological systems while being kept within boundaries^10–23, 55–57^. At the genome levels, stochasticity characterizes many processes parallel to the deterministic pathways^58^. Similarly, multiple stochastic effects underlie the standard processes at the protein, cells, and tissue levels^59, 60^. Noise characterizes whole organ function, including the heart, blood pressure, respiratory system, gait, brain functions, and immune system^61–69^. The CDP defines diseased states as associated with too much or too low degrees of noise in cases where the noise is unconstrained or where the boundaries are too tight. Based on this notion, the use of noise to improve systems function was proposed^10, 55–57^.

Second-generation AI systems dynamically implement personalized variability signatures^26^. It implies looking at the variability of clinical parameters (heart rate variability, respiratory variability), laboratory testing (genetic variability, cytokine secretion variability), and variability of proteomics, metabolomics, and lipidomics^26–28^. These signatures are dynamic, and so is the degree of variability, mandating a continuous adaptation of the models to improve the outcome of the interventions.

CDP-based second-generation AI platforms implement noise into medical interventions for improving clinical outcomes. The use of this system for overcoming tolerance to chronic drugs and improving efficiency was suggested^29, 70–88^. In patients with heart failure and diuretic resistance, implementing variability into the treatment regimen improved the heart failure-related symptoms, functional capacity, and heart failure-related laboratory tests. Significant reductions in emergency room admissions and hospitalizations due to heart failure were noted during the intervention^89^. Similarly, improved clinical response was noted by implementing noise into the treatment regimens in patients with multiple sclerosis and subjects suffering from chronic pain^57^. Implementing variability in training and exercising may improve performance^29, 90^. Similar to the use of this method for overcoming drug tolerance, it can overcome the plateau effect in training where the brain-muscle-nerves connections prohibit the ability for continuous improvement^90^. The present study’s data endorse the notion that variability can improve the efficacy of non-pharmacologic anti-aging interventions, such as PA, supported by the CDP

It was previously suggested that implementing variability into training regimens may overcome the neuromuscular, cardiopulmonary, and hormonal adaptation mechanisms that result in the plateau effect, facilitate recovery, and improve performance^29^. This hypothesis primarily addresses professional athletes who perform high-intensity sports. Several studies imply that lower intra-individual variability is associated with poorer function. For instance, using fractal analysis techniques to generate variability-based indices from wearable sensors showed that mortality and aging were associated with reducing the complexity of daily PA patterns^42^. Additional studies have demonstrated reduced PA complexity among patients with Alzheimer’s disease^43^ or frail individuals^91^. Therefore, reduced variability in PA patterns may serve as a biomarker of aging and frailty. Other studies have demonstrated that highly variable or fragmented PA patterns, as determined by minute-to-minute analysis of continuous accelerometry monitoring, may also be associated with frailty and mortality^92–94^. They concluded that these patterns may be an early sign of physiological decline.

Aging is a multifactorial process and is the subject of multiple interventional studies aiming to halt or reduce its progression and alleviate age-related disorders^95^. It is an irreversible and gradual pathophysiological process. Various aging-related diseases are associated with decreased tissue and cell function, including neurodegenerative diseases, cardiovascular diseases, metabolic diseases, musculoskeletal diseases, and immune system diseases^95^. Research on aging examines how endogenous and exogenous stresses, genomic instability, cellular senescence, telomere dysfunction, epigenetic alterations, compromise of autophagy, mitochondrial dysfunction, stem cell exhaustion, altered intercellular communication, and deregulated nutrient sensing play a role in aging regulation^95^. Age-related sarcopenia is characterized by loss of muscle mass, sedentary habits, and negative protein balance^96^.

Variability is associated with aging. Associations between variability and hallmarks of aging and the roles of disorder and variability of systems in the pathogenesis of aging were described^87^-transcriptional variability increases in response to immune stimulation with aging^97^. Older adults are more likely toexhibit variable performance due to either attention or intention lapses. Older adults have higher response time with more variability than young adults. However, if the differences in speed between older adults and young adults are taken into account, the older adults appear to be less variable than the young adults. Response time (RT) and intraindividual variability were positively correlated. Both within-subjects and between-subjects (slower individuals were more variable regardless of age) showed the same relationship (practice increased speed and reduced variability). According to the data of a previous study, average response times may be generally correlated with performance variability, and older adults’ more significant performance variability is primarily due to their greater average response times^98^.

An extensive cohort of subjects was studied for mean age differences and variability in episodic memory and executive function measures. Over adulthood, cognitive performance declined rapidly in the early 60s on associative recognition, spatial working memory, speed, facilitation, and set-shifting - a shift from gradual to rapid decline. Individual variability (interindividual variability or diversity) and intraindividual variability (dispersion) also increased gradually until the 60s and rapidly thereafter^99^. There is more significant moment-to-moment variability in blood oxygen level-dependent (BOLD) signals (SDBOLD) in various brain cortical regions among younger, better-performing adults. The dynamic range may be reduced in the aging brain. It has been demonstrated that vascular factors contribute to differences in fixation-based SDBOLD among younger adults, with younger adults exhibiting higher SDBOLD. The variability of BOLD signals persists after comprehensive control of vascular effects, suggesting that it is an essential marker of brain aging^100^. Older adults have more motor variability than young adults, which affects their accuracy and function. Age-related variability can be reduced by low-intensity training that emphasizes muscle coordination. A low amount of visual feedback minimizes age-related differences in variability. Old age is associated with a change in gait variability^101^.

Variability in PA regimens of non-athlete adults may be intentional or constrained, as they are subjected to daily obligations or physical disabilities. Several factors may contribute to the beneficial effects of the variability. First, by varying the frequency of PA, active adults may overcome adaptation mechanisms and reduce the risk of overuse injuries associated with repetitive exercise. This method can be especially beneficial among aging workers prone to repetitive strain work-related injuries^102^. Second, inactive adults may have different schedules, commitments, or physical limitations that affect their ability to engage in frequent exercise. By incorporating variability into training regimens, individuals can maintain a sustainable routine that allows them to reap the benefits of PA while accommodating their individual needs. Last, from a CDP-inspired perspective, an adequate level of variability recognized as an inherent property of the system may be necessary for the system’s normal function and directly result in outcome optimization. This approach implies that regaining the physiological complexity may pave new therapeutic avenues.

Per the CDF, physiological processes with too much or too low degrees of variability may accelerate the aging process. Based on this concept, implementing variability into interventions in old subjects was suggested to improve their efficacy^24^. The present study’s data support the premise that using second-generation AI systems that implement variability into intervention may improve the results. Implementing a CDP-based second-generation AI system for improving anti-aging modalities was recently proposed^87^. The platform uses constrained noise to enhance systems’ efficiency and slow the aging process. The present study’s data support suggests that this system can slow down aging-related processes.

This study provides novel insights to health professionals who employ PA programs for non-athlete adults in general and within the workplace. Inactivity is a worldwide pandemic, and variability in PA regimens may hold promise in improving the health outcomes of infrequently active individuals. These variability-implemented regimens may result in better health outcomes and be better tolerated by the “average” irregularly active adult. Our results also reinforce the claim that reduced variability among inactive adults may indicate functional deterioration, necessitating further assessment and interventions.

Although it has some strengths regarding the sample size, background demographic, health data, long follow-up, data reliability emanating from repeated harmonized panel data collection, and generalizability, our study has some limitations that must be addressed. First, the inherent disadvantages of self-reported data include recall bias and confounders. These limitations were diminished by data acquisition methods (i.e., multiple choice of broad and distinct categories of PA frequencies guided by a qualified interviewer) and multivariate analysis. Second, causal relations cannot be unequivocally concluded in an observational study. We attempted to augment the temporal relations between variability in PA and consequent disability by allocating the last available wave exclusively for outcomes measurement and using only the early follow-up waves for PA intensity analysis, as aforementioned. However, studies are needed to confirm these results, and whether variability in PA frequency is a marker or the source of physical disability emergence remains unsolved. Last, we could not reliably quantify the overall amount of PA in the *stablevs. unstable PA* subgroups, so we cannot disprove the argument that the superior outcomes in the *unstable PA* subgroup among inactive adults are purely the result of a higher total PA load. Nonetheless, the sustained statistically significant improvement in the subgroup analysis of individuals with the same (low) PA frequency at the start and end of the follow-up (Fig. 2B) eliminates the possible health effects of persistently high PA intensity.

In summary, the present study’s data support that introducing variability into interventions may attenuate aging processes. Variability in PA frequency may be significant for the health of inactive or less physically active adults after the age of 50. By incorporating variability into their PA rituals, non-athletes can tailor their exercise habits to fit their lifestyle and maintain a balanced routine that may be associated with improved physical and cognitive outcomes. Future studies may investigate other variability aspects of PA, such as duration, intensity, and short-term (e.g., day-to-day) variations, which could not be extracted from our dataset. Furthermore, the effects of variability on frail individuals, its impact on psychological outcomes, and the impact of different variability-based regimens should be examined. Studies are planned to determine the possibility of implementing variability-based second-generation AI tools that introduce personalized interventions based on dynamic variability signatures.

## Figures and Tables

**Table 1.** Study subject’s characteristics.

Variable	N	Stable, N = 513^1^	Unstable, N = 1,536^1^	p-value^2^
**Gender**	2,049			>0.9
male		291 (57%)	870 (57%)	
female		222 (43%)	666 (43%)	
**Age at baseline**	2,049	53.5 (3.8)	54.0 (3.9)	0.021
**Diabetes melitus**	2,047	22 (4.3%)	69 (4.5%)	0.8
**Stroke history**	2,048	4 (0.8%)	7 (0.5%)	0.5
**Ischemic heart disease history**	2,048	11 (2.1%)	42 (2.7%)	0.5
**COPD history**	2,048	12 (2.3%)	22 (1.4%)	0.2
**PA intensity at Baseline**	2,049			<0.001
High		434 (85%)	1,039 (68%)	
Low		79 (15%)	497 (32%)	
**Same PA intensity at start and end**	2,049			<0.001
Same		386 (75%)	501 (33%)	
Varying		127 (25%)	1,035 (67%)	
**Short term memory at baesline**	2,039	5.74 (1.55)	5.71 (1.58)	>0.9
**Long term memory at baesline**	2,041	4.40 (1.80)	4.36 (1.82)	0.6
**Verbal fluency at baesline**	2,033	24 (7)	23 (7)	0.2

1 n (%); Mean (SD)

2 Pearson’s Chi-squared test; Wilcoxon rank sum test; Fisher’s exact test

## Abbreviations

PA: physical activity
CDP: constrained-disorder principle
AI: artificial intelligence
SHARE: survey of health, aging, and retirement in Europe
ADL: activities of daily living
RT: response time
BOLD: blood oxygen level-dependent
SDBOLD: blood oxygen level-dependent signal variability
